# NICU and postpartum nurse perspectives on involving fathers in newborn care: a qualitative study

**DOI:** 10.1186/s12912-021-00553-y

**Published:** 2021-02-23

**Authors:** Katharine W. Buek, Dagoberto Cortez, Dorothy J. Mandell

**Affiliations:** 1 University of Texas Health Science Center at Tyler & Population Health, Office of Health Affairs, University of Texas System, 210 W. 7th St., Austin, TX 78701 USA; 2 Department of Sociology, The University of Texas at Austin, 305 East 23rd St., A1700, Austin, TX 78712-0118 USA

**Keywords:** Father engagement, Father involvement, Mother-baby nursing, Perinatal care

## Abstract

**Background:**

Perinatal care nurses are well positioned to provide the education and support new fathers need to navigate the transition to fatherhood and to encourage positive father involvement from the earliest hours of a child’s life. To effectively serve fathers in perinatal settings, it is important to understand the attitudes, beliefs, and behaviors of healthcare providers that may encourage and engage them, or alternatively alienate and discourage them.

**Methods:**

This qualitative study involved structured interviews with ten NICU and postpartum nurses from hospitals in two large Texas cities. The interview protocol was designed to elicit descriptive information about nurses’ attitudes and beliefs, sense of efficacy and intention for working with fathers, as well as their father-directed behaviors. Nurses were recruited for the study using a purposive sampling approach. Interviews were conducted by telephone and lasted approximately 25 to 35 min. Data were analyzed using a qualitative descriptive approach.

**Results:**

Overall, study participants held very positive subjective attitudes toward fathers and father involvement. Nevertheless, many of the nurses signaled normative beliefs based on race/ethnicity, gender, and culture that may moderate their intention to engage with fathers. Participants also indicated that their education as well as the culture of perinatal healthcare are focused almost entirely on the mother-baby dyad. In line with this focus on mothers, participants comments reflected a normative belief that fathers are secondary caregivers to their newborns, there to help when the mother is unavailable.

**Conclusions:**

Nurse attitudes and practices that place mothers in the role of primary caregiver may be interpreted by fathers as excluding or disregarding them. Further research is needed to validate the results of this small-scale study, and to assess whether and how provider attitudes impact their practices in educating and engaging fathers in newborn care.

**Supplementary Information:**

The online version contains supplementary material available at 10.1186/s12912-021-00553-y.

## Background

Perinatal care nurses are well positioned to provide the education and support new fathers need to navigate the transition to fatherhood and to encourage positive father involvement from the earliest hours of a child’s life. Many nurse education programs and hospitals have adopted family-centered models of care as the nominal standard for the provision of perinatal services. According to the principles of family-centered care (FCC), the provider’s role is to provide service in accordance with the needs and wishes of women and their families to the extent possible, educate parents so that they can make informed decisions about care, and involve and empower parents to provide care for their child in the hospital and at home. However, the translation of FCC principles from theory to action has been slow and uneven across care contexts [1]. Additionally, definitions and implementation of FCC principles can vary substantially across healthcare organizations, hospitals, and even units within a single hospital. Evidence suggests that adherence to the principles of FCC may also vary from provider to provider, according to their training, personal views, and experience [2].

In fact, despite the increasing focus on family and its importance in the provision of quality health care, healthcare systems and providers often neglect and sometimes alienate fathers^1^ [3–7]. Fathers have reported feeling “excluded” and “invisible” in perinatal care settings, relegated to a secondary role in which their own needs and perspectives are not always valued by providers [4]. In many cases, information and educational content focuses entirely on mothers. No information is provided to fathers about their own role and providers may not even look at or speak directly to fathers [5]. Such practices are at odds with the goals of FCC and of perinatal care to educate and empower parents to care for their own children.

A first step in improving the experience of fathers in perinatal settings is to better understand the knowledge, attitudes, and practices of the health care providers who interact with them. Existing studies of nurse attitudes suggest that nurses may hold biased views that disadvantage fathers. In a Swedish study, the nurses believed fathers were competent caregivers to their infants and that they should be involved in both caregiving and attending CHC visits. Still, 65% agreed with the statement “mothers are instinctively better at caring than fathers are” [8]. Another Swedish study concluded that child health nurses “do not see it as their core competency or task to actively promote father involvement” [9]. As of the time of writing, there were no studies of healthcare provider attitudes toward fathers conducted in the United States, and none which explored provider-father interactions in perinatal care settings. Nurses’ attitudes and beliefs about fathers and fathering, as well as their perceptions of their own role and competencies, may affect the quality of care they provide to fathers. This study sought to explore the knowledge, attitudes, and practices of nurses who interact with fathers on a daily basis. The ultimate aim of this work is to identify opportunities for education, training, or quality improvement initiatives to improve the perinatal care experience for fathers and families.

## Methods

### Protocol development

**Fig. 1:**
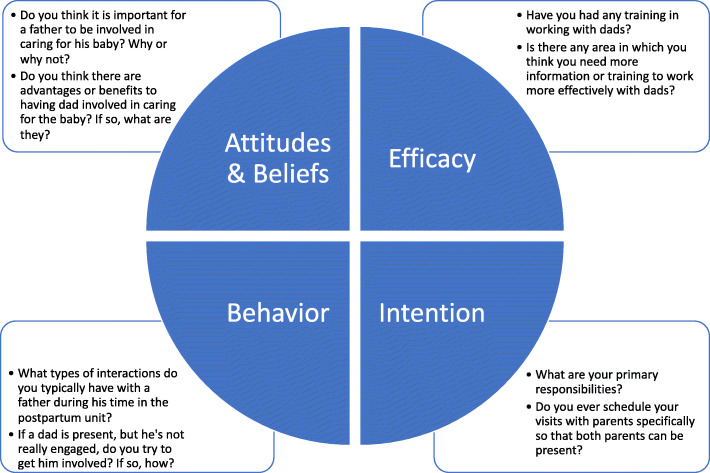
Example questions addressing nurse attitudes & beliefs, efficacy, intention and behavior related to father engagement

This qualitative study was carried out as part of a larger study of behavior change among postpartum nurses in hospitals across Texas. The Theory of Planned Behavior (TPB) [10] served as the theoretical framework for the study. TPB has been used extensively in the study of behavior change in health contexts and has frequently been employed in research on nurse attitudes and practices. The interview protocol was designed for the purposes of this study to elicit descriptive information about nurses’ attitudes and beliefs, sense of efficacy and intention for working with fathers, as well as their father-directed behaviors. The protocol contained 19 questions asking about nurses’ perceived role and responsibilities, training received, how they approach and engage fathers, and their perceptions of fathers’ role and involvement in newborn care. Figure 1 displays examples of interview questions addressing each of the components of TPB. The interview protocol is included as Supplementary Material.

### Sample

Nurses were recruited for the study using a purposive sampling approach. Study coordinators distributed information about the purpose and parameters of the study via email to colleagues with established relationships with postpartum units in area hospitals. Nurses who were interested in participating in the study were asked to contact study coordinators to express interest. Criteria for participant selection included current or recent prior experience working in postpartum (mother-baby) units. Fifteen nurses contacted the research coordinator to express interest in participating in the study. All of these nurses met study criteria, however due to budget constraints, only ten were selected to participate. Nurses were selected purposively to represent as many different hospitals as possible, including those serving both private and public payer populations. Interview participants included ten nurses – nine female and one male – working in postpartum units in four hospitals located in two large Texas cities. At the time of data collection, five nurses were working in postpartum units and three in the NICU. One participant was a nurse navigator with prior experience in both postpartum and NICU and one worked as an education resource specialist with prior experience as a postpartum nurse.

### Data collection and analysis

This study employed a qualitative descriptive (QD) approach to data collection and analysis. In contrast with other qualitative frameworks, the QD approach is primarily concerned with describing phenomena (e.g. who does what, how things are done, feelings and perceptions), rather than developing theory or providing in-depth interpretations of experience [11]. A QD approach is especially useful in exploring topics that are not well researched and for research designed to inform the development of survey questionnaires [12]. Additionally, this approach allows for identification of themes in the data as well as quantitative analysis of responses in terms of frequencies of particular responses [13].

The first author conducted all interviews by telephone; each lasted approximately 25 to 35 min. Written consent was waived so that participants could not be connected to their responses. It was important that participants felt secure in discussing the dynamics of their work environment without fear of reprisal by their employers. The purpose and goals of the research (i.e. to better understand nurse experiences with fathers) were explained briefly prior to the interview and verbal consent was obtained prior to the start of the interview. Detailed fieldnotes were compiled during each interview. These fieldnotes were further developed using analytic memos right after interviews and later during the formal analytic process [14, 15]. Participants’ responses were entered into REDCap (Research Electronic Data Capture), a web-based software application that securely stores survey and other data in an easy-to-use format [16]. Upon completion of the interviews, participants received $150 electronic gift cards as compensation for their time.

Data coding and analysis were done in NVivo version 12 [17]. Both conventional content and thematic analyses [18, 19] were applied to interview responses in this study. Responses were first grouped by interview question, such that each question initially served as the unit of analysis. Within questions, an inductive approach to coding was employed [20]. After detailed coding at the question level, a second round of coding identified themes that cut across multiple questions. Both latent and manifest content of the interview responses was analyzed. Finally, themes and codes were grouped into the broad categories of nurse role and training, interactions with fathers, fathers’ engagement in newborn care and education, and perceptions of fathers and the father role. Table 1 shows the final coding structure with categories, themes and codes. Questions are not shown because responses often cut across multiple categories and themes. Saturation was reached for key codes and themes, as nurse responses to most questions were highly consistent.

**Table 1:** Final coding structure with categories, themes and codes

Category	Themes	Codes
Nurse role and training	Direct care of mother, baby	Assessment Cord and circumcision care Diapering, bathing, swaddling Feeding Post-operative care
	Parent education	Mother relays info to father Repeat information for father Teach whomever is present Wait for father to arrive
	Focus on mothers	Training focuses on mothers Education directed to mothers
	Want, need additional training	Communication Fathers’ interests, concerns Providing education How to engage fathers
Interactions with fathers	Father presence or absence at hospital	Home with other children Incarcerated Not involved with the mother Out of country Transportation issues Work schedule, lack of leave
	Ways to engage fathers	Answer his questions Ask about his thoughts, feelings Demonstrate, show him things Directly acknowledge him Don’t push too hard Educate and inform him Encourage him to ask questions Tell him he needs to learn, do things
Perceptions of fathers’ level of engagement	Level of engagement	As engaged as mothers Less engaged than mothers
	Influences on level of involvement	Depends on the person First-time fathers more engaged Cultural influences
	Barriers to involvement	Afraid to touch or handle the baby Feels left out, ignored Lack of knowledge, confidence Maternal gatekeeping
Attitudes toward father involvement	Benefits of father involvement	Benefits to baby Benefits to mother Benefits to family
	Father role	Equal partner with mother Secondary caregiver

## Results

Interviews revealed details about when and how the participant nurses interact with fathers in the postpartum unit, as well as broader perspectives and assumptions they held toward fathers and fathering.

### Nurse role and training

The participants described their primary responsibilities in terms of direct care to the mother and newborn, including mother and newborn assessments and breastfeeding instruction. In addition to direct patient care, the nurses unanimously reported that educating parents was a key part of their job. They noted that while the education they provide is not specifically targeted to fathers, they attempt include him in their efforts when he is present. Participants repeatedly mentioned teaching to whomever is present in the room. If the father is not present at the time information is provided, the nurses reported that they either return to repeat the information later or rely on the mother to relay information to him. None of the participants reported scheduling their visits or timing educational interactions for when both parents can be present; one noted “It has never been a consideration. Either they can be there, or they can’t. You have to get people when they’re there.” Mothers served as the gatekeepers for most of the information and education the nurses provided.

The participants acknowledged that postpartum education primarily targets mothers. A veteran nurse specifically noted that this emphasis on mothers is a direct product of nurse training. She explained, “As a younger nurse, I was told that if I’ve taught the mom, then I’ve done my job.” Another suggested that nurses might benefit from additional training in how to engage fathers “because [fathers] may have points of view that we don’t consider because we are trained to take care of mom.” One nurse with several years of experience specifically noted that broader systems change is needed saying, “Institutionally, a lot of pressure is placed on the mom. There’s a lot of tacit acceptance that this is the way it is.”

Nurses in the study had not received training specific to working with fathers or how to engage them in parent education and newborn care. Most did not feel that they needed any additional training, saying that on-the-job experience was sufficient. Some participants suggested they would benefit from additional training in such topics as fathers’ concerns, interests and learning needs; how to get fathers more involved; and fathers’ mental and emotional states following a birth. Some indicated that training in communication with fathers would be beneficial. One female nurse noted that it is “easier to communicate with women mother-to-mother, but harder with fathers.” Another felt that it would be good to learn how to respectfully approach fathers who might be reluctant to get involved.

### Interactions with fathers

A primary goal of the interviews was to understand nurses’ approaches to educating and involving them in newborn care. All participants reported that when fathers are in the room, they try to include him in what they are teaching or doing. From responses to multiple questions about nurse interactions with fathers, several different approaches were coded (see Table 2). These approaches ranged from explaining and demonstrating aspects of newborn care to more personal approaches like directly acknowledging fathers, providing encouragement and positive feedback, and eliciting fathers’ thoughts and feelings. The most commonly employed techniques were explaining/informing and suggesting ways fathers could help with routine aspects of care. Personal and direct approaches were more rarely mentioned and represented a concerted effort on nurses’ part to establish rapport and bring the father into the nurse’s sphere of operation.

**Table 2:** Nurses’ approaches to engaging fathers, code categories and examples

Code	Examples
Answer/elicit fathers’ questions (*n* = 4)	“The ones that are interested stand around watching what you do, and I’ll answer questions”
	“They are receiving so much information and are so stressed because the baby is in serious condition, but we encourage them to ask questions.”
Explain things to, inform him (*n* = 10)	“They kind of come hover and ask, ‘Why are you doing that?’ and ‘What’s that for?’ I’ll let them stand and watch and explain why they do that.”
	“At discharge, we bring out … extra information on postpartum depression, we go over it with them, like instructions on feeding, car seats, stuff for taking the baby home.”
Directly acknowledge him (*n* = 8)	“If you want to and are motivated to, you can make eye contact, but it requires making effort.”
	“If you get dads on the first day, acknowledge them and communicate expectations for them both, they will get involved.”
Demonstrate, show him how to do things (*n* = 5)	“If they are awake I try to talk to him to see what he knows about diapers, feeding. I’ll say, ‘Let’s try while I’m here so I can give you some pointers’.”
	“Telling them, ‘Come change this diaper’ can be off-putting. ‘Come help me change this diaper, I’ll show you what to do’ is more useful.”
Suggest ways he can help (*n* = 10)	“I feel out the situation to see what his involvement is, and I’ll say, ‘Hey, Dad, do you want to help out with the bath?”
	“That’s when we address dads directly instead of mom, like say ‘Mom’s been changing the diaper, do you want to come do it?”
Offer encouragement, positive feedback (*n* = 3)	“I’ll have dad help wash the baby… and I give him positive feedback for the first bath. Try to get them as involved as possible.”
	“You have to take their hand, and in a gentle, encouraging way get them to do things.”
Ask about his thoughts, feelings (*n* = 3)	“I try to get them to open up and discuss their feelings and stress, explain what’s happening.”
	“When you’re having a conversation with both parents say ‘Dad, what do you think?’ asking him directly instead of just letting mom answer.”
Tell him he needs to learn, do things (*n* = 4)	“I will try to get them to change diapers and I’ll say, ‘This is your baby, this baby is not coming home with me.’”
	“If it’s something really important, especially outpatient, I might say, ‘Hey dad, you really need to pay attention to this.’”

Note: *n* = number of times the code was applied across all nurse interviews, multiple mentions by a single nurse were coded as separate instances

One participant suggested that the level of effort she expends to involve fathers depends on her initial impressions of the father’s engagement, saying:

Especially as a bedside nurse you can tell how helpful they’re going to be. If they seem interested, I’ll include them more. If they don’t seem to be interested, I’ll ask but I don’t make them help…. Sometimes you interact more with them because they act like they are going to be more helpful.

All participants indicated ways in which they try to involve fathers but noted that they do not push the issue if a father seems uninterested or unmotivated. Several times, they indicated that when engaging with fathers they are careful not to “pressure them.” The nurses mentioned “feeling out” fathers’ level of interest and attempting to involve them without imposing on personal, family or cultural values (see Table 2 for examples).

### Perceptions of fathers’ level of involvement

In addition to questions about the ways that they interact with and involve fathers, the nurses were asked about their perceptions of fathers’ level of engagement. These questions addressed fathers’ level of interest, attention, and participation in various aspects of education and newborn care. Responses to these questions reflected a belief that fathers’ involvement depends largely on individual, relational, and cultural factors. The following comment summarizes these perceptions: “Really every dad is so different. Some are really involved, and some will sleep through the whole day, and some are not interested at all.” Another nurse put it this way, “different people, different backgrounds… it varies from couple to couple. It could be cultural or the situation.” Almost all participants referenced culture in their interviews. One noted that Nigerian fathers tended to be very engaged and ask a lot of questions, and that young African American fathers were often as engaged and involved as the mothers. Several nurses noted that fathers of Latin American, Middle Eastern, South Asian, and West African backgrounds typically expect the mother to assume the role of caring for the newborn.

Participants acknowledged that there were few resources or services available for fathers. One nurse noted that when her own children were born, her husband “felt like there was nothing geared towards him.” Another nurse recounted an incident in which a father became upset because he felt the hospital staff “were disregarding him, or the doctors were not taking him seriously.” The nurses frequently noted that hospitals offered classes, support groups, or other activities for mothers, but did not have equivalent supports for fathers. One example was provided by a NICU nurse who mentioned that the unit held special events for Mother’s Day, but not for Father’s Day.

### Attitudes and beliefs regarding father involvement

Participants were emphatic in stating that it is important for fathers to be involved in caring for their newborns, with most indicating that this is “definitely” or “absolutely” important. The nurses believed that father involvement was beneficial for the child, especially in terms of bonding and forming a strong father-child relationship. They also noted benefits to the mother, in terms of reduced stress and depression and benefits for family functioning.

In analyses, two opposing views of a father’s role emerged. Sometimes fathers were described as equal partners with the mother, and other times as a secondary caregiver – a “relief hitter” for the mother (Table 3). “Father as secondary caregiver” was coded more than twice as often as “father as equal partner.” Sometimes this theme was explicit, as when one nurse noted that “We do try to encourage [fathers], tell them they may need to do it while mom’s not available.” Other times it was implied, as when one nurse noted that it’s important for fathers to be involved because “mom needs a break once in a while.” The “father as secondary caregiver” theme also applied in the hospital context, such as when a participant noted that fathers were sometimes asked to perform skin-to-skin care with the newborn when the mother’s temperature was too low.

**Table 3:** Alternative views on fathers’ caregiving role, code categories and examples

Code	Examples
Father as equal partner (*n* = 6)	“[Fathers] are one of the parents and it shouldn’t just be mom…The father is just as important in the family relationship.”
	“It takes a village. They dynamics of what a mom does versus a dad, it’s different… [Children] need the support of both parents.”
	“I treat dad just like I treat mom, because they are both caregivers.”
	“Yes, because he’s a caregiver too. Just because mama gave birth, it’s his responsibility as much as hers.”
Father as secondary caregiver (*n* = 13)	“[Fathers can] seek opportunities to get involved, like if the baby starts crying when mom is in the shower, what can he do.”
	“I always say it’s important to help your wife as much as possible, because taking care of a baby is hard...”
	“He can’t breastfeed, but mom can do that, and dads do diapering and stuff… [to] relieve mom of her duties.”
	“Mom may feel more at ease, because if she has to go back to work, then dad can stay home with the baby. It’s important for mom to know she’s supported by dad and he knows what he’s doing in her absence.”

Note: *n* = number of times the code was applied across all nurse interviews, multiple mentions by a single nurse were coded as separate instances

## Discussion

The findings of this study have important implications for current conceptualizations of family-centered maternity care. Recent decades have seen important shifts from heavily provider-driven models of perinatal care to models that give women and families more control over their pregnancy, birth, and postpartum care. Nevertheless, postpartum care as currently conceptualized and implemented remains focused primarily on women and children [21, 22]. While the nurses in this study held positive views about fathers and fathering generally, these did not necessarily translate into an intentional effort to engage and educate fathers.

TPB provides a framework in which to consider how attitudes and normative beliefs, including efficacy, positively or negatively influence an individual’s intention to carry out a goal-directed behavior [10]. In the case of this study, the target behavior is engagement with fathers for the purpose of educating and empowering them to care for their newborns. Nurses in this study demonstrated ambivalence toward engaging fathers, with mixed and sometimes competing views on fathers and fathering, as well as their own role and capacity to engage with fathers.

### Attitudes, intention, and behavior

Attitudes and beliefs have been shown to predict, to a significant degree, whether individuals will engage in a particular behavior [23]. First, there are attitudes about the behavior itself, such as whether the behavior is good or bad, useful or not useful, etc. Second, there are normative beliefs, or subjective norms, which involve perceptions of social pressure either to perform or not perform the behavior. Subjective norms include the perceived opinions and expectations of significant members of the individual’s social circle, including family members, friends, co-workers, and employers [23]. Finally, behavior is predicted by an individual’s assessment of his or her perceived behavioral control or efficacy; only to the extent that a person believes they have the ability to perform the behavior will they form an intention to carry out the behavior and carry it out.

Nurses in this study held positive, if biased, attitudes regarding father involvement. They were unanimous in stating that fathers should be involved in the care of their infants at home. However, participant comments reflected a normative belief that mothers held primary responsibility for infant care, and fathers were there to step-in when she was unavailable or needed rest. Regarding attitudes toward engaging fathers in the hospital context, participants indicated that parent education was a key aspect of their role, though attitudes or intentions toward interacting with and educating fathers were not expressed. Father involvement seems to be incidental to what they perceive as their primary role – caring for mothers and newborns.

Beliefs about behavioral control were similarly ambivalent. None of the nurses had received any training in working with fathers – some specifically noted that their training exclusively focused on the mother-baby dyad – and yet most felt their education and training were adequate for working with fathers effectively. They were also clearly concerned about overstepping individual, family and cultural boundaries, which they view as the primary determinants of a fathers’ role and involvement in newborn care. Participants expressed a normative belief that whether and how fathers are involved in caring for the newborn is up to the couple and the family according to their personal and cultural preferences. This view may reflect an attitude that involving fathers is not only outside the nurse’s role, but also beyond her behavioral control.

The nurses describe various ways that they try to engage fathers in newborn care and education. However, they do not appear to have a clear motivation, intention, or strategy for doing this effectively. In their own words, they “try their best” to involve fathers as much as is expected, given an institutional environment that prioritizes mothers. They engage fathers in the best way they know how, given a lack of specific training or tools for working with them. And they do it as unobtrusively as possible to avoid overstepping familial and cultural boundaries. If fathers are to receive the support they need from perinatal care systems, it will be necessary to reshape providers' attitudes and intentions around father involvement.

### Implications for perinatal nurse education and practice

The findings of this study are largely consistent with studies carried out in Europe and Canada [4, 8, 9, 24]. These studies also found that perinatal health care providers viewed fathers positively but as supporting actors to the mother as primary patient, caregiver, and parent. A qualitative interview and document review study conducted in Canada also found little attempt to include fathers by perinatal health care providers [4]. Interviews revealed that there were few educational or other services for fathers, and document reviews found that when fathers were explicitly mentioned, it was primarily as part of the mother’s support network. Additionally, the document review showed that nurses recorded twice as many observations on mothers as fathers and four times more interventions with mothers. Significantly, these findings were published some 15 years after the introduction of Canada’s National Guidelines for Family-centered Maternity and Newborn Care [25].

To date, the literature on “family-centered” maternity care remains focused on the experiences and outcomes of mothers and infants, largely to the exclusion of fathers [21, 22, 25–28]. Family-centered maternity care interventions typically emphasize mother-child interaction and women’s satisfaction, while evaluations of family-centered evaluations rarely assess outcomes for fathers or the father-child bond [29–33]. Research and practice in the NICU have made strides toward including fathers with research exploring fathers’ specific needs, concerns, and mental and emotional states, as well as strategies for increasing their participation in care [34–39]. More research is needed to understand fathers’ unique needs for information and support during the perinatal period and how this can be delivered effectively. Such research can be used to inform systemic change in perinatal care, including policies and institutional protocols that acknowledge fathers as equal with the mother; education and training for health care providers to meet fathers’ needs effectively; and evidence-based tools to be used in hospital and community-based contexts.

### Limitations

It is important to acknowledge certain limitations to the conclusions that can be drawn from this study. First, the sample is small and not representative of the population of postpartum nurses. The nurse interviews conducted here were designed to inform the development of a questionnaire to be employed in a much larger survey of nurses throughout the state. This study was intended to offer an initial exploratory look into nurses’ interactions with fathers and the attitudes that might shape those interactions. It was determined that a small convenience sample of nurses from different hospitals in two of the state’s major population centers would suffice for this purpose.

Second, the recruitment procedures used in the study allow for the possibility of self-selection bias in the data collected. Nurses were told that a study was being conducted about attitudes related to father involvement in postpartum unit and were asked to contact researchers if they were interested in participating. It is possible that the nurses who volunteered for the study are more positive and proactive toward father involvement than those of the total population. Nevertheless, we feel that the study uncovered some important biases in attitudes and practices that merit further investigation. Additional research with larger and more representative nurse samples should explore these questions.

## Conclusion

Changes to the perinatal health care system that bring the father into greater focus could improve the healthcare experiences of fathers and families. Family-centered perinatal care should acknowledge both parents as the primary caregivers for their infant and recognize that mothers and fathers may have different, but equally important, needs and desires for support in the transition to parenthood. Further research is needed to explore the changes in provider attitudes and practice that may be necessary to improve fathers’ experiences with perinatal care. The primary aim of postpartum nursing must be to ensure that both mothers and fathers receive the best possible information and support to help them embrace their roles as parents in the hospital and as they transition to the home environment.

## Supplementary Information

**Additional file 1.** Structured Nurse Interview Protocol. Interview protocol listing all questions asked of nurse participants during the interview.

## Data Availability

The datasets used and/or analysed during the current study are available from the corresponding author on reasonable request.

## Abbreviations

CHC: Community Health Center
FCC: Family-centered Care
NICU: Neonatal Intensive Care Unit
QD: Qualitative Descriptive
TPB: Theory of Planned Behavior

## Footnotes

1 For the purposes of this discussion, the term “father” refers to any adult male who has responsibility for providing care and support to the child, whether he is a biological or adoptive parent, the mother’s married or unmarried partner, or any other type of “father figure” in the child’s life.
