# Supplementary material for: NICU and postpartum nurse perspectives on involving fathers in newborn care: a qualitative study
Source: BMC Nurs. 2021 Feb 23;20:35. doi: 10.1186/s12912-021-00553-y (PMC7903796; doi:10.1186/s12912-021-00553-y)
Supplement: Supplementary file 1 — Additional file 1. Structured Nurse Interview Protocol. Interview protocol listing all questions asked of nurse participants during the interview. [file 12912_2021_553_MOESM1_ESM.docx]

**Nurse Structured Interview Protocol**

Welcome, thank you for meeting with me today. I am from Safe Babies project atThe University of Texas Health Science Center Tyler. We are working on an evaluation of a program that is designed to get fathers more involved in caring for their babies.

The program involves providing information to dads in the mother-baby units, and we will be collecting info from dads, but would also like to get nurses’ perspectives. Specifically, we are interested in understanding how nurses interact with dads in the hospital, their perceptions of dads’ involvement and understanding of neonatal and post-partum issues, and barriers to educating or involving dads.

I will be conducting interviews like this with nurses from different hospitals, with different specialties and different levels of experience. Anything we discuss will be confidential, not discussed outside the research team, and reports will not identify specific individuals or hospitals, so no one will know who said what.

There are no right or wrong answers to these questions. The objective of this interview is simply to gain an understanding of what nurses do and think about their interactions with new parents, particularly related to new dads and their roles in caring for their babies. These issues can sometimes be sensitive or even controversial, but it is important to know the reality of what happens in the hospital so that programs can be designed to address these realities effectively.

Can we proceed with the interview?

By responding “yes,” you are giving your consent for your responses to be used by the Safe Babies team. All responses are confidential. Your name will not be recorded.

Yes No

INTERACTIONS WITH MOM AND DAD (est. 5 minutes)

1. What unit(s) do you work in?
2. What are your primary responsibilities?
3. What is your first encounter with a new mother who is transferred into your unit? (describe)
   1. Probe: And the father? (describe)
4. What types of interactions do you typically have with a mom during her time in the mother-baby/NICU unit?
   1. Probe: What about dads?

IF DAD IS NOT PRESENT (est. 5-10 min)

1. Are dads usually present in the unit when you are interacting with a new mom?
   1. Probe: What are some of the reasons a dad might not be present?
2. If a dad is not present, do you have any way to know if the mom has a relationship with him?
   1. Probe: Do you ask the mom any questions about the dad? Explain why or why not.
   2. Probe: Do you speak with the mom about getting the dad involved?
3. If a dad is not present during a particular visit when you provide important information or education, what do you do?
4. Do you ever schedule your visits with parents specifically so that both parents can be present?

ENGAGING DADS (est. 10 min)

1. Are there aspects of your job in which dads participate along with moms? Please describe.
   1. Probe: Aspects in which dads are usually not involved?
   2. When dads are present, would you say they are not as engaged, as engaged, or more engaged than moms during the times you are providing information/assisting them? Please explain.
   3. Probe: Do dads pay attention when you are interacting with the mom? Probe: Do they ask you questions?
   4. Probe: Do they share their thoughts or concerns with you?
2. If a dad is present, but he’s not really engaged, do you try to get him involved? If so, how?
3. Have dads ever expressed to you any concerns about not being as involved as they might want to be? Please describe.
4. Some dads might not feel comfortable learning about how to care for a baby, or believe that it’s more appropriate for the mom to do this. Have you ever experienced this perception in dads that you have worked with?
5. Do you think there is anything you could do, in your job, to help get these dads more involved?
6. Have you had any training in working with dads, the kinds of concerns they have, or the things they are most interested in learning?
7. Is there any area in which you think you need more information or training to work more effectively with dads?

DADS’ ROLE IN CARING FOR BABY (est. 5-10 min)

1. Do you think it is important for a dad to be involved in caring for his baby? Please explain.
   1. Probe: What types of things do you think dads should do?
2. Do you think dads are less capable, as capable, or more capable in caring for their babies than moms? In what ways?
3. Do you think there are advantages or benefits to having dad involved in caring for the baby? If so, what are they?
   1. Probe: What about disadvantages or risks associated with dads caring for the baby?
4. Is there anything else you would like to share with me with regard to your experiences or perceptions of your work with fathers?

THANK YOU FOR TAKING THE TIME TO SPEAK WITH ME. YOUR AMAZON GIFT CARD WILL BE EMAILED TO YOU WITHIN THE NEXT FEW DAYS. IS THIS THE EMAIL ADDRESS WHERE YOU WOULD LIKE TO RECEIVE YOUR GIFT CARD? [CONFIRM EMAIL ADDRESS ON RECORD.]
